# Does delayed measurement affect patient reports of provider performance? Implications for performance measurement of medical assistance with tobacco cessation: A Dental PBRN study

**DOI:** 10.1186/1472-6963-8-100

**Published:** 2008-05-08

**Authors:** Thomas K Houston, Joshua S Richman, Heather L Coley, Midge N Ray, Jeroan J Allison, Gregg H Gilbert, Judith S Gordon, Catarina I Kiefe, The DPBRN Collaborative Group

**Affiliations:** 1 Deep South Center on Effectiveness Research, a VA HSR&D REAP, Birmingham VA Medical Center, Birmingham, AL, USA; 2 Division of General Internal Medicine, University of Alabama at Birmingham, Birmingham, AL, USA; 3 Center for Outcomes and Effectiveness Research, University of Alabama at Birmingham, Birmingham, AL, USA; 4 Division of Preventive Medicine, University of Alabama at Birmingham, Birmingham, AL, USA; 5 Department of Health Services Administration, University of Alabama at Birmingham, Birmingham, AL, USA; 6 Department of Diagnostic Sciences, School of Dentistry, University of Alabama at Birmingham, Birmingham, AL, USA; 7 Oregon Research Institute, Eugene, OR, USA

## Abstract

**Background:**

We compared two methods of measuring provider performance of tobacco control activities: immediate "exit cards" versus delayed telephone follow-up surveys. Current standards, e.g. HEDIS, use delayed patient measures that may over or under-estimate overall performance.

**Methods:**

Patients completed exit cards in 60 dental practices immediately after a visit to measure whether the provider "asked" about tobacco use, and "advised" the patient to quit. One to six months later patients were asked the same questions by telephone survey. Using the exit cards as the standard, we quantified performance and calculated sensitivity (agreement of those responding yes on telephone surveys compared with exit cards) and specificity (agreement of those responding no) of the delayed measurement.

**Results:**

Among 150 patients, 21% reporting being asked about tobacco use on the exit cards and 30% reporting being asked in the delayed surveys. The sensitivity and specificity were 50% and 75%, respectively. Similarly, among 182 tobacco users, 38% reported being advised to quit on the exit cards and this increased to 51% on the delayed surveys. The sensitivity and specificity were 75% and 64%, respectively. Increasing the delay from the visit to the telephone survey resulted in increasing disagreement.

**Conclusion:**

Patient reports differed considerably in immediate versus delayed measures. These results have important implications because they suggest that our delayed measures may over-estimate performance. The immediate exit cards should be included in the armamentarium of tools for measuring providers' performance of tobacco control, and perhaps other service delivery.

## Background

Quality assessment using discreet performance measures is rapidly expanding in clinical practice. Defining measures and choosing the method for quantifying performance is an increasingly high-stakes activity as quality assessment become the basis for incentive pay-for-performance and other marks of prestige [1-3]. Specific criteria for choosing appropriate performance measures have been published. These criteria suggest focusing on measuring health care services that are related to important health conditions, relevant to a large number of patients, have varied performance, and have a strong evidence base for performance [4]. Tobacco use and related health services such as brief provider cessation counseling clearly fit these criteria.

Around 438,000 premature deaths are caused by cigarette smoke in the United States each year [5]. Systematic screening and brief clinical interventions have proven effective in helping patients quit tobacco [6]. Based on this literature, a goal of Healthy People 2010 is to have 85% of providers perform such counseling about smoking cessation to patients [7].

The Health Plan Employer Data and Information Set (HEDIS), a set of standardized performance measures collected by the National Committee for Quality Assurance, has adopted patient-reports of provider tobacco cessation advice as a national standard. In 1996 HEDIS began asking smokers whether a plan provider advised them to quit smoking in the last year [8]. The initial rates showed 61% of physicians in managed care plans advised patients with the rate increasing to 65% in 2002 [8]. HEDIS is used by over 90% of health plans in the United States for various performance measures. The current tobacco control performance measures are quantified yearly using a mixed-methods approach based on mail and telephone surveys to assess performance at visits that occurred in the past year.

Unfortunately, these assessments may suffer from limited or faulty recall due to the delay from clinical visit to follow-up assessment. Measuring patient-reported outcomes and behaviors as close to the targeted exposure as possible has been recommended [9-11]. In the context of a larger group-randomized trial to improve tobacco control amongst dentists, we developed a brief "exit card" survey that was distributed by dental practice staff in 60 dental practices and completed by patients immediately following the clinical appointment, while still in the dental office.

The study was designed to assess the feasibility of distribution of the exit cards, and then compare the variation in a HEDIS-like assessment (delayed phone survey) with the immediate assessment (exit card) of the same patients in sixty no-intervention (control) dental practices. The performance of practices and individual providers are increasingly being compared using standardized performance measures. Movements such as pay for performance are based on performance data [12,13]. Practices and providers can be rewarded financially if they are top-performing or potentially penalized if they are below a certain threshold for a variety of performance measures. Thus, we conducted this additional analysis to estimate the impact of immediate versus delayed on performance ranking.

## Methods

### Study design

This study is a sub-study using baseline data from a randomized trial among dental practices from Alabama, Georgia, Florida, and North Carolina. The randomized trial identified practices using dental licensure lists and mailing lists from the "Dental PBRN", a dental practice-based research network [14]. Dental practices were recruited using a letter to the dentist advertising the study. For blinding purposes the letter did not mention tobacco control but identified the study as an evaluation of an "Online Study Club for Oral Cancer Prevention." Eligible practices included general dentistry or periodontal practices reporting having Internet access in their practice and indicating interest in participating. Briefly, the randomized trial intervention was an Internet-delivered continuing education intervention designed to increase rates of tobacco cessation advice provided by dental providers to their patients. Once a practice agreed to participate, the dentist assigned a contact person at the practice to be responsible for data collection and interaction with the study coordinating center.

This study in this report compares two methods of patient-report of provider tobacco control performance – immediate "exit cards" and delayed telephone surveys. For this study, we recruited patients from the first 60 practices from the larger study to obtain sufficient precision to compare immediate and delayed assessment of receipt of tobacco cessation advice by providers. Because we did not wish to confound the findings of this sub-study with any potential effects of the larger randomized trial intervention, we included only non-intervention practices in these analyses. The protocol was approved by the University of Alabama at Birmingham Institutional Review Board.

### Data collection – Patient exit cards (immediate assessment)

The patient exit cards, brief post-card sized surveys, were completed by adult patients at the end of their appointments prior to leaving the office. The exit cards were developed using principles of ecological momentary assessment (EMA), [9-11] a method used in health behavior and more recently in health services research to overcome limitations of traditional self-report assessments [9,11,15]. First, EMA is completed as close in time to the exposure as possible to avoid limited or faulty recall. Second, EMA is designed to be brief and unobtrusive to maximize participation rates and diffusion.

To develop the exit cards, three investigators with expertise in tobacco control and survey design identified potential questions. Then the exit cards were reviewed by our full panel of investigators and an external consultant. The cards were then pilot tested in our academic dental practice. For the pilot, 29 cards were distributed by front office staff and 24 cards were returned with questions completed (response rate = 83%). Patients were asked to record the time required to complete the pilot survey. The average time to complete was two minutes, and ranged from one to three. We then revised the cards based on data collected and comments and recommendations made by patients and dental staff.

Each practice was provided a set of 100 patient exit cards for consecutive distribution. The practice was instructed to hand out exit cards to a hundred consecutive patients immediately at the end of their visit. Each patient was offered a ball-point pen with the study logo to complete the survey, and to keep as an incentive. Patients deposited completed exit cards in a sealed collection box in the practice, typically at the reception desk. If a patient was not interested in completing the exit card, they were instructed to write "decline" on the card and return the card to the box. After all 100 exit cards were distributed, the dental practice sent the collection box to our coordinating center.

The patient exit cards were used to assess whether the patients were tobacco users, as well as their age and gender. We also asked whether the patients had been asked during that day's dental visit about tobacco use (referred to below as "ask"), and, if they were a tobacco user, whether they had been advised to quit (referred to as "advise"). To blind the patient and practice to the outcome of interest, the exit card also included questions related to the patient's alcohol and dietary intake and whether they received counseling related to alcohol use and dietary habits. Finally, patients were asked if we had their permission to call them later for a telephone survey and if so, they were asked to provide their name, telephone number, and best day/time to call.

### Data collection – Patient phone survey (delayed assessment)

We contacted patients who provided their name and telephone number to complete a second, delayed assessment. Participants in the delayed telephone surveys were provided a $10 gift card as reimbursement for their time. Blinded to patients' previous immediate exit card report of "ask" and "advise" (yes or no) and provider overall performance, trained interviewers asked the same "ask" and "advise" questions from the exit card, again referring to the index visit.

#### Delayed assessment of "Ask"

Because all patients, both tobacco users and non-tobacco users, are eligible for "ask," we wanted to compare immediate versus delayed assessment for all patients. Because a greater number of patients were eligible for "ask," we were able to compare the two assessments with reasonable precision from a smaller number of practices. Data collected from both tobacco users and non-tobacco users in six practices from January 10 through March 30, 2006 (6 week period) were used to assess "ask". The same patients from these six practices also completed the six month telephone follow-up assessment. These practices were selected to represent a range of follow-up intervals from one to six months to further assess the impact of time delay on agreement. The interviewers asked patients about their dental visit when the exit card was completed, and used the same question to assess "ask" that was asked on the exit card.

#### Delayed assessment of "Advise"

Because a smaller number of patients per practice (only smokers) were eligible for "advise," we needed a larger number of practices to compare the immediate and delayed with precision. We contacted tobacco users who completed the immediate exit cards from 60 practices by telephone six months after the immediate data collection to assess point prevalence tobacco cessation, a primary outcome of the study.

### Analysis

Demographic characteristics unlikely to be subject to limited or faulty recall (age, gender) and also tobacco use status were compared using the immediate exit cards and delayed telephone surveys. Agreement rates for provider performance – ask and advise – using the exit cards and telephone surveys were also compared. Based on prior reports that immediate patient reports agree reasonably well, compared with a gold standard of audiotapes, we also calculated sensitivity and specificity of the delayed telephone survey, when considering the immediate exit card as a standard. For "ask," because follow-up time varied from one to six months, we also assessed the proportion of ask, and agreement with the exit cards, stratified by follow-up time.

Finally, for "advise," we assessed how using the exit card versus the telephone survey affected the relative performance ranking of practices. Patient exit card and telephone survey data were aggregated at the practice level. Performance was defined as the proportion of tobacco users who reported receiving advice to quit tobacco. Using these data, we ranked providers into quartiles of performance based on data from the immediate exit card data, and then again using data from the delayed telephone surveys.

## Results

In the 60 practices, 6,000 exit cards were distributed (100 per practice) and overall response rate was 80% (4,776/6,000). Of these, 21% (1,018/4,776) were tobacco users. These general dentistry practices were mostly solo practices (82%) with a mean of two hygienists per practice. These characteristics were similar for the six practices targeted for the "ask" analysis.

### Assessment of screening for tobacco use ("Ask")

In the six practices used to assess "Ask," the exit card response rate was 520/600 (86%). Patients had a mean age of 45 (SD 14) years, 67% were female, and 19% were tobacco users. Based on the exit cards, only 25% (132/520) of patients reported that the dental staff had asked them if they were tobacco users. This rate of ask in the six practices was similar to that (28%) in all 60 practices.

Of the 520 exit card completers, 208 patients agreed to be contacted for follow-up. There were no differences in demographics comparing these 208 to the overall sample of respondents from these six practices. Of the 208 patients from six practices contacted for delayed telephone surveys regarding "ask", 150 (72%) completed the survey. Mean time from immediate exit card (date cards were returned to our office) to follow-up call completed was three months (SD 1, range 1–6 months). Agreement on whether the patient was a tobacco user was high (99%), with only two disagreements. Of these two disagreements, one participant reported having quit, but being a tobacco user at the time of the visit and the other reported being a non-tobacco user at baseline, but a user on follow-up. Agreement rates for patient age and gender comparing immediate and delayed were also high (97% and 100%, respectively).

Agreement on whether providers asked patients about tobacco use was lower (Table 1). Measured performance for "ask" changed from 21% using the immediate exit cards to 30% using the delayed telephone survey method. Overall agreement for "ask" was 70%. Of those at baseline saying "Yes, I was asked by the dentist or dental staff if I use tobacco", 50% (16/32) again reported "Yes" at follow-up. Conversely, of those at baseline saying "No, I was not asked by the dentist or staff if I use tobacco", 75% (89/118) responded with a "No" at follow-up. Thus, if we consider the exit card as a reference, the sensitivity and specificity of the follow-up were 50% and 75%, respectively. Age was not related to agreement, but women had a higher rate of agreement (77%), compared with men (50%), chi-square = 10, p = 0.001.

**Table 1:** Patients' reports of "Ask"* on immediate exit cards versus delayed telephone surveys

		**Immediate exit card**	
	
Telephone survey @ 1–6 months later		Did the dentist or dental staff ASK you if used tobacco?	
	Total N = 150	**Yes **N = 32 (21%)	**No **N = 118 (79%)
Did the dentist or dental staff ASK you if used tobacco?	**Yes **N = 45 (30%)	16	29
	**No **N = 105 (70%)	16	89
		Sensitivity = 50% (95% CI 31%, 68%)	Specificity = 75% (95% CI 66%, 82%)
		Agreement 105/150= 70% (95% CI 62%, 77%)	

* Ask (Patients' report of Tobacco Use Screening from providers): 150 patients from six dental practices, the DTC.net study, 2006

When divided by quartile of follow-up time, measured performance for "ask" by telephone survey increased with length of delay between index visit and follow-up telephone survey: 22% of patients reported they were asked for first quartile of follow-up time (less than 1.5 months), 25% for second quartile, 31% for third quartile, and 41% for longest quartile of follow-up (over 3.5 months) (p for trend = 0.02). Patients with the longest follow-up, over 3.5 months, had the lowest overall agreement (56%) with the immediate exit cards, with those in other quartiles similar at 75% (p = 0.03).

### Assessment of quit tobacco advice ("Advise")

Of the tobacco users who completed exit cards in the 60 practices, 254 agreed to be contacted for follow-up and provided a valid telephone number. We were able to contact 194 (74%) of these tobacco users, and 182 answered the "advise" on the immediate and delayed assessments. These tobacco users were mostly female (57%) with mean age of 44 years (SD 13). The proportion of the 182 tobacco users advised to quit based on exit cards was 38% (95% CI 31%–46%) and on delayed phone assessment was 51% (95% CI 44% – 59%). Agreement was 68% and sensitivity and specificity were 75% and 64% respectively (Table 2). Immediate versus delayed agreement did not vary with gender or age.

**Table 2:** Tobacco users' reports of receipt of "Advise"* on immediate exit cards versus delayed telephone surveys

		**Immediate Exit Card**	
	
Telephone Survey @ 6 months later		Did the dentist or dental staff ADVISE you to quit using tobacco?	
	Total N = 182	**Yes **N = 70 (38%)	**No **N = 112 (62%)
Did the dentist or dental staff ADVISE you to quit using tobacco?	**Yes **N = 93 (51%)	53	40
	**No **N = 89 (49%)	17	72
		Sensitivity = 75% (95% CI 63%, 85%)	Specificity = 64% (95% CI 55%, 73%)
		Agreement 125/182= 68% (95% CI 61%, 75%)	

* Advise (tobacco user's receipt of tobacco cessation advice from providers) on immediate exit cards versus delayed telephone surveys at six months: 182 tobacco users from 60 practices, the DTC.net study, 2006

### Change in quartile rank of performance for advice to quit tobacco

Patient-reported provider performance on the immediate exit cards (baseline) was compared to the delayed telephone surveys to show how practice performance rankings on the "advise" question changed from baseline to telephone follow-up. Patient data for tobacco users was collapsed at the practice level. Overall practice performance rankings for "advise" was similar on the immediate exit cards (mean proportion advised = 43% (SD 36)) compared to the delayed phone calls (mean proportion advised = 39% (SD 32), paired t-test of mean proportions p = 0.22). However, 49% of practices changed rank comparing the delayed telephone assessment-based ranking versus rankings based on the immediate exit cards (Table 3). As noted in Table 3, of the top performing practices (Quartile 4) at baseline, 40% dropped by one or more quartile rankings in the delayed assessment. We repeated this analysis limiting to a sample of practices with over five patient reports per practice (a more stable estimate of practice performance) and found similar results.

**Table 3:** Change in practice performance ranking quartiles: comparing practice performance ranking based on tobacco users' report of provider performance (advice to quit) on immediate exit card assessment versus delayed telephone assessment.

	Change in Performance based on Delayed Telephone Assessment		
	Performance Ranking Increased by one or more quartiles	Performance Ranking Unchanged	Performance Ranking Decreased by one or more quartiles

Practice Performance Ranking based on Immediate Exit Card Assessment*	n/N (%)	n/N (%)	n/N (%)
Fourth Quartile (N = 15) (Top Performers)	N/A	9/15 (60%)	6/15 (40%)
Third Quartile (N = 14)	3/14 (21%)	5/14 (36%)	6/14 (43%)
Second Quartile† (N = 16)	6/16 (38%)	8/16 (50%)	2/16 (12%)
First Quartile (N = 15) (Lowest Performers)	6/15 (40%)	9/15 (60%)	N/A
Overall (N = 60 Practices)	15/60 (25%)	31/60 (52%)	14/60 (23%)

* Practice Performance Ranking Quartiles based on proportion of patients from the practice reporting advice to quit (immediate exit card quartiles: First Quartile = 0% to 19% of tobacco users from the practice reporting advice; Second Quartile = 20% to 33% reporting advice; Third Quartile = 34% to 75% reporting advice; Fourth Quartile = 76% to 100% reporting advice).

† Second Quartile includes 16 practices due to a tie in ranking

## Discussion

For the two measures of provider performance used in this study (i.e. "ask" and "advise"), delayed assessment was found to over-estimate overall performance when compared with immediate assessment by exit cards. Agreement was moderate, with sensitivity and specificity of the delayed measures varying from 50% to 75% as compared with the immediate measures.

At the practice level, many who were "top performers" based upon immediate assessment fell to lower quartiles of rank on the delayed assessment. Performance measures will always have limitations. Our analysis adds to a growing body of literature on patient-reported performance measurement. We found that the timing of the performance assessment mattered. Because some practices' relative performance ranking varied considerably based on immediate versus delayed measurement, consumers of performance rankings, such as the U.S. Center for Medicare and Medicaid Services (CMS), need to carefully consider whether such measures are stable enough to serve as a basis for financial reward or penalization.

We speculate that some of the difference in immediate versus delayed assessments relate to the way memories are stored. Long-term memory of past experiences does not fully decode details in unbiased ways [16]. Although recall of memories can produce believable and reasonable results, the results may or may not represent an accurate account of the target situation [17]. When patients are uncertain about their answer they will often say "yes" rather than "no", possibly as a mechanism to give the "right" answer and "cover" for the physician [18]. Thus, in quality assessment, the use of memory is especially a concern regarding patient recall of a clinical visit that occurred in the past. To avoid the problems of memory, a recent National Institutes of Health conference on measurement advocated newer methods, such as ecological momentary assessment, [9-11] to measure patient-reported outcomes and behaviors as close to the targeted exposure as possible.

Although our study focused on comparing time delays in patient assessments, prior studies have also evaluated other sources of quality assessment data for tobacco cessation counseling including provider reports and chart abstraction. Provider self-report often over-estimates performance and may be influenced by the Hawthorne effect (observing behavior may alter it) [19]. Chart abstraction is expensive and may underestimate provider performance for tobacco control because tobacco cessation counseling is often not documented in patients' charts [20]. In fact, compared to the gold standard of direct observation (audio or videotapes of doctor-patient encounters), immediate surveys of patients have been previously shown to be more accurate than provider reports and sometimes chart abstraction [20-22]. Prior studies have questioned the validity of using other methods such as provider reports and chart abstraction for performance assessment related to behavioral counseling and specifically tobacco-related screening and advice [19-24].

In contrast to costly methods such as chart abstraction, our patient exit cards were feasible to implement at relatively low cost (approximately $80 per practice), much lower than the cost of the gold standards for performance measurement of provider counseling for tobacco control, i.e., direct observation, standardized patients, or even chart abstraction. In this study, we demonstrated the willingness of practices to distribute and collect patient exit cards, and 86% of patients in participating practices were willing to complete the exit cards. We used patient exit cards, distributed by office staff, in a prior single-site study with a similarly high response rate (over 80%) [25]. This rate is considerably higher than rates expected from a mailed survey or a cold-call telephone interview.

This study has several limitations. One is that we do not have a "gold standard," such as audio/videotape or standardized patients, to use as a reference for the validity of these reports. The gold standard of traveling to a practice to perform in-person evaluations with direct observation was not feasible for a study of this size with dental practices across the Southeast. Because the agreement rate declined with time for "ask," and in view of previously published reports, the immediate patient assessment is more likely to represent a true reflection of the patient experience. Further study is needed to verify if the exit cards accurately reflected what happened in the clinical encounter. Of note, the observed differences in rates could be due to the "method" as well as the time sequence. Social desirability bias is greater for telephone calls versus paper-based surveys [26,27]. Thus, social desirability and time lag may have conspired to increase the rates of performance comparing telephone to exit cards.

A second limitation is this study was conducted in only one patient population and with only two measures. Our population sample was limited to dental patients in five Southeastern states and may not be generalizable to all dental practices and patients. Also, the performance measures used by this study were not identical to the HEDIS measure, making the two measures less comparable. HEDIS asks about all visits over a specified time period however, our survey asks only about the visit where the exit card was completed. Asking about the same visit was intended to make the delayed assessment comparable to the immediate assessment. We speculate that questions asking about a broader time period may suffer even more from limited or faulty recall, but this was not addressed in our study. Additional studies are necessary to determine the generalizability of this patient population and measure, especially to directly compare with measures used by HEDIS.

An additional limitation is that we are dependent on the practices to distribute and collect the exit cards. This introduces the risk of biased selection of patients to complete the exit cards, or even falsification of data. We instructed the practices to distribute the cards to 100 consecutive patients to avoid biased sampling. Of note, the overall rate of tobacco use in the sample was approximately what we would expect to see based on the population. Also, the high agreement rate for variables such as age, gender, and tobacco use status provides evidence that practices did not falsify data. Although participation rates in our follow-up survey were high, it is possible that loss to follow-up biased our estimates of agreement in some way. Furthermore, some individuals completing the exit cards did not agree to be contacted for a delayed telephone survey, thus again raising the potential for bias. However, there is no reason to believe that patients declining follow-up would have had more accurate recall at follow-up. Therefore, our main finding of delayed recall accuracy should not be influenced by the refusal rate. We included only dental practices in this study, and thus results may not be generalizable to all medical providers.

## Conclusion

We documented that delayed surveys did not agree with patient-reported performance measurement conducted immediately at the time of the clinic visit. Our results have important implications because they suggest that delayed measures may over-estimate or under-estimate performance. Further, this study found disagreement increased as time elapsed from visit to telephone survey. Combined with higher accuracy in immediate reports from other studies, our findings suggest that immediate assessment may provide a different, potentially more accurate result than delayed assessment. Because widely used and accepted quality measurement systems such as HEDIS use telephone surveys querying patient recall over an entire year, our findings may have significant implications for the assessment of tobacco control in clinical practice.

Immediate ecological momentary assessment using patient exit cards was acceptable to patients and providers in a large sample of clinical practices, with high response rates (86% of patients were willing to complete the cards). The exit card should be included in the armamentarium of tools for measuring providers' performance of tobacco control, and perhaps other service delivery.

## Competing interests

The authors declare that they have no competing interests.

## Authors' contributions

TH- Principle Investigator of the study, conducted the data analysis with the oversight of JR, drafted the initial manuscript with HC, and reviewed and approved the final draft. JR, HC, MR, JA, GG, JG, and CK participated in study design and data collection, critically reviewed, edited, and approved final draft.

## Pre-publication history

The pre-publication history for this paper can be accessed here:


